# Application of mesenchymal stem cell-derived exosomes from different sources in intervertebral disc degeneration

**DOI:** 10.3389/fbioe.2022.1019437

**Published:** 2022-10-07

**Authors:** Yuanliang Xia, Ruohan Yang, Yulin Hou, Hengyi Wang, Yuehong Li, Jianshu Zhu, Changfeng Fu

**Affiliations:** ^1^ Department of Spine Surgery, The First Hospital of Jilin University, Changchun, China; ^2^ Cancer Center, The First Hospital of Jilin University, Changchun, China; ^3^ Department of Cardiology, Guangyuan Central Hospital, Guangyuan, China

**Keywords:** extracellular vesicles, intervertebral disc degeneration, mesenchymal stem cells, cartilage endplate stem cells, extracellular matrix

## Abstract

Intervertebral disc degeneration (IVDD) is a main cause of lower back pain, leading to psychological and economic burdens to patients. Physical therapy only delays pain in patients but cannot eliminate the cause of IVDD. Surgery is required when the patient cannot tolerate pain or has severe neurological symptoms. Although surgical resection of IVD or decompression of the laminae eliminates the diseased segment, it damages adjacent normal IVD. There is also a risk of re-protrusion after IVD removal. Cell therapy has played a crucial role in the development of regenerative medicine. Cell transplantation promotes regeneration of degenerative tissue. However, owing to the lack of vascular structure in IVD, sufficient nutrients cannot be provided for transplanted mesenchymal stem cells (MSCs). In addition, dead cells release harmful substances that aggravate IVDD. Extracellular vesicles (EVs) have been extensively studied as an emerging therapeutic approach. EVs generated by paracrine MSCs retain the potential of MSCs and serve as carriers to deliver their contents to target cells to regulate target cell activity. Owing to their double-layered membrane structure, EVs have a low immunogenicity and no immune rejection. Therefore, EVs are considered an emerging therapeutic modality in IVDD. However, they are limited by mass production and low loading rates. In this review, the structure of IVD and advantages of EVs are introduced, and the application of MSC-EVs in IVDD is discussed. The current limitations of EVs and future applications are described.

## 1 Introduction

Intervertebral disc degeneration (IVDD) is among the most common spinal degenerative diseases and the main cause of chronic low back pain (LBP) in clinical patients (Zhang et al., 2021a). Approximately 40–50% of clinical LBP cases is caused by disc degeneration (Geurts et al., 2018). IVDD is a process of aging and damage caused by a series of complex molecular mechanisms and usually caused by extracellular matrix (ECM) breakdown and anabolic disturbances, leading to IVD bulging and loss of water content in the nucleus pulposus (NP) and subsequent loss of disc height (Kirnaz et al., 2022). IVDD causes partial or complete rupture of the annulus fibrosus (AF); the NP protrudes backward from the rupture and irritates or compresses the nerve root, thus causing low back and leg pain (Adams et al., 2015). LBP seriously endangers the physical and mental health of patients and usually results in a serious economic burden. In the United States alone, the cost of LBP treatment exceeds $100 billion per year (Katz, 2006). The prevalence of IVDD is increasing yearly due to risk factors, such as aging, obesity, chronic stress, and smoking (Kirnaz et al., 2022).

Clinical treatment for IVDD mainly includes drug therapy, bed rest, traction, acupuncture, massage, and electromagnetic or electrothermal therapy (Xin et al., 2022). Although these conservative treatments may relieve symptoms and reduce pain, they do not reverse IVDD (Oichi et al., 2020). Patients who cannot tolerate pain usually undergo surgical removal of the IVD or IVD fusion therapy (Xin et al., 2022). Surgical treatment eliminates the radicular pain caused by IVDD, as it fundamentally eliminates the source of LBP. However, surgical resection of the IVD may lead to reherniation, and reherniation rate after lumbar disc herniation was as high as 21.2% (Ran et al., 2015). In the long term, IVD fusion eliminates the mobility of adjacent cones, increasing the load and stress on the surrounding tissues and IVD, which leads to degeneration of other IVDs in adjacent segments (Kumar et al., 2001). Many therapeutic strategies for IVDD have been developed in tissue engineering and regenerative medicine. However, these modalities are only in the preclinical stage.

**SCHEME 1:**
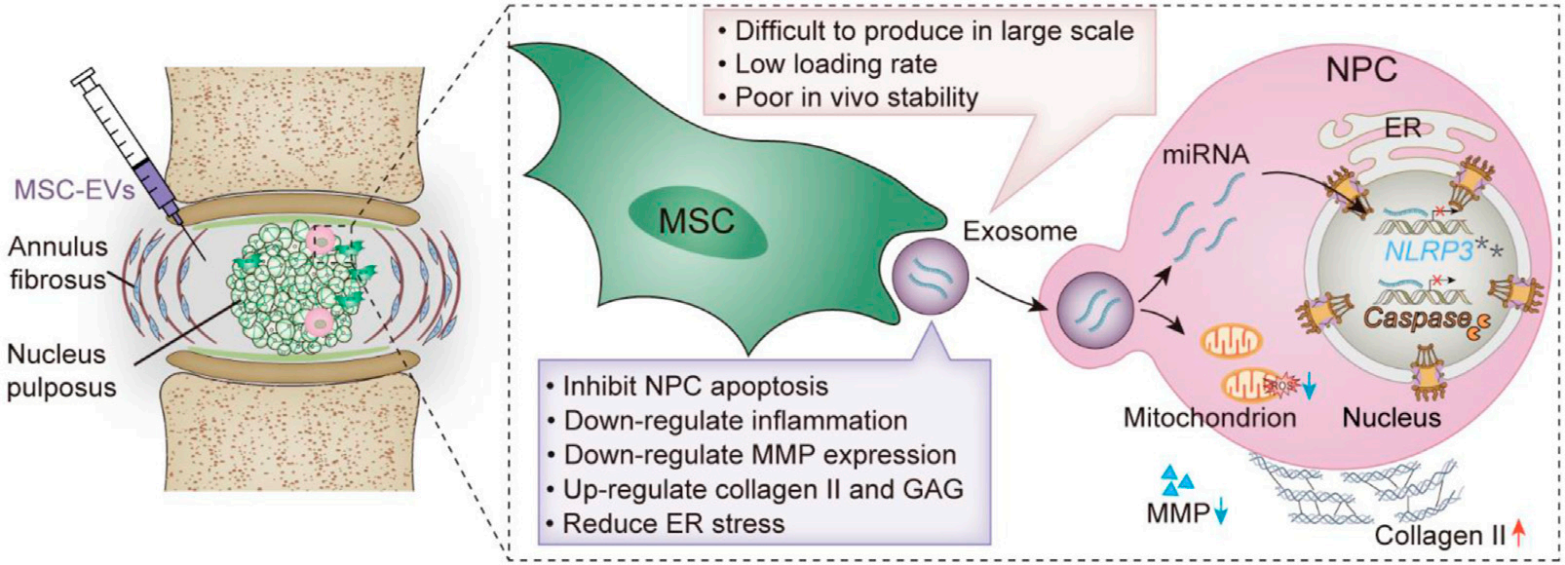
Schematic illustration of MSC-EVs promoting IVDD repair. Advantages and disadvantages of MSC-EVs.

With advances in cell therapy, stem cell repair of IVDD appears to be a good strategy. Based on the tissue engineering, stem cells have been widely used in regenerative medicine owing to their advantages of self-proliferation and directed differentiation at degeneration sites (Moeinabadi-Bidgoli et al., 2021). Mesenchymal stem cells (MSCs), induced pluripotent stem cells (iPSCs), and adipose stem cells can differentiate into NP cells (NPCs) and synthesize new ECM for IVDD regeneration (Baldia et al., 2021; Ekram et al., 2021). Moreover, stem cells also reduce the inflammatory environment around IVDD through paracrine secretion of anti-inflammatory factors and chemokines (Zhang et al., 2022). However, the IVD is an avascular structure; the stem cells delivered around the IVDD cannot absorb enough nutrients from the surrounding tissue and cannot fully exert their effect of regenerating the NP and AF (Hajiesmailpoor et al., 2022). Stem cells delivered to the area surrounding IVDD die in large numbers due to the inability to take up nutrients; the inflammatory factors [interleukin (IL)-1, IL-6, and IL-8] released by dead cells will also cause surrounding inflammatory responses, which are usually not conducive to the survival of surrounding cells (Yuan et al., 2021a).

With the development of tissue engineering and regenerative medicine, extracellular vesicles (EVs) have made great progress as active biological substances for intercellular communication owing to their ability to promote tissue regeneration (Wang et al., 2022). MSC-EVs have therapeutic potential for the treatment of IVDD by promoting cell proliferation and tissue regeneration, modulating inflammatory responses, and reducing apoptosis (Bhujel et al., 2022). EVs originate from intracellular multivesicular bodies (MVBs), vesicular structures that are released from cells in a paracrine fashion into the extracellular matrix. EVs have a bilayer lipid membrane structure and have the advantages of low immunogenicity and good cytocompatibility *in vivo*. EVs contain cytokines, proteins, lipids, and non-coding RNAs. The transfer of EV content between cells inhibits the apoptosis of NPCs and promotes ECM synthesis to delay IVDD (Wang et al., 2022). In addition, EVs circumvent the adverse effects of stem cell therapy for IVDD, making it a promising therapeutic strategy for IVDD (DiStefano et al., 2022). In this review, the composition of IVDD is first introduced. Second, the generation and advantages of EVs and their applications in IVDD are discussed (schematic illustration and Table 1). Finally, the limitations of EV applications are mentioned, and future research directions are enumerated.

**TABLE 1:** Mesenchymal stem cell-derived extracellular vesicles in intervertebral disc degeneration.

Source of EVs	Size	Deliverables	Target	Results	References
BMSCs	50–130 nm	Mitochondrial-associated protein	NPCs,ECM	EVs inhibited the activation of the NLRP3 inflammasome and attenuated the apoptosis of NP cells and decreased MMP-13 expression in the ECM.	Xia et al. (2019)
	30–150 nm	Cavin-2	NPCs	Cavin-2-engineered EVs restored the impaired endocytic pathway of NPCs and enhanced the therapeutic effect of regenerating NPCs	Liao et al. (2021)
	30–150 nm	-	NPCs	BMSCs-EVs relieved compression-mediated NPCs apoptosis by inhibiting oxidative stress and mitochondrial damage	Hu et al. (2021)
	94.3 nm	-	NPCs	MSC-EVs can attenuate ER stress-related apoptosis during IVDD.	Liao et al. (2019)
	100 nm	-	NPCs	MSC-EVs attenuate apoptosis by enhancing the autophagy of NPCs by promoting the Akt-mTOR signaling pathway	Xiao et al. (2022)
	40–100 nm	miR-155	NPCS	BMSC exosomes increase the expression of miR-155 in NPCSs, which targets Bach1 and then upregulates HO-1 expression, activates autophagy in NPCSs, and inhibits the level of apoptosis	Shi et al. (2021)
	78 ± 8 nm	miR-21	NPCs	Exosomal miR-21 inhibits PTEN, thereby activating the PI3K/Akt pathway in apoptotic NPCs	Cheng et al. (2018)
	80 nm	miR-142-3p	NPCs	Exosome-packaged miR-142-3p can target MLK3 to inhibit MAPK signaling	Zhu et al. (2020a)
	110 nm	circ_0072464	NPCs	EVs-transferred circ-0072464 upregulates NRF2 expression through competitive binding to miR-431, and its mechanism leads to inhibition of NPC ferroptosis and alleviation of IVDD.	Yu et al. (2022a)
	80 nm	microRNA-199a	NPCs	MiR-199a carried by BMSCs-EVs promotes IVDD repair by targeting GREM1 and downregulating the TGF-β pathway	Wen et al. (2021)
	30–150 nm	circ_0050205	NPCs	BMSC-EVs carrying circ-0050205 promote NPC survival and attenuate IVDD progression by regulating the expression of miR-665 and GPX4	Yu et al. (2022b)
	30–150 nm	microRNA-129-5p	NPCs,ECM	BMSC-derived EVs transfer miR-129-5p to NP cells and perturb macrophage apoptosis, ECM degradation, and M1 polarization by targeting LRG1 and inactivating p38 MAPK signaling	Cui and Zhang, (2021)
	60–200 nm	microRNA-194-5p	NPCs	MSCs-EVs overexpressing miR-194-5p ameliorate the pathology of IVDD by inhibiting TRAF6	Sun et al. (2022)
	109.3 nm	miR-532-5p	NPCs,ECM	Exosomes from BMSCs alleviate the progression of IVDD by preventing NPC apoptosis, ECM degradation and fibrotic deposition via miR-532-5p	Zhu et al. (2020b)
PLMSCs/UCMSCs	30–150 nm	AntagomiR-4450	NPCs	hPLMSC-EVs carrying AntagomiR-4450 alleviate IVDD and improve gait impairment by upregulating ZNF121	Yuan et al. (2020)
	65 ± 15 nm	miR-26a-5p	NPCs	UCMSC-EVs deliver exogenous miR-26a-5p to suppress pyroptosis in NPCs via METTL14/NLRP3	Yuan et al. (2021b)
	-	-	ECM	UCMSC-EVs have the potential to alleviate ECM degradation through the p38 MAPK pathway	Qi et al. (2019)
ADSCs	40–200 nm	-	NPCs	ADSCs-EVs increased NPCs migration and proliferation and suppressed inflammatory activity	Zhang et al. (2021c)
	30–150 nm	dECM@exo	ECM	dECM@exo has the ability to modulate inflammatory complexes and metalloproteinases	Xing et al. (2021)
iPSCs	80–200 nm	miR-105-5p	NPCs	iMSC-sEVs exert their anti-aging effects by delivering miR-105-5p to senescent NPCs and activating the Sirt6 pathway	Sun et al. (2021)
NPCs	282 nm	FOXF1	NPCs,ECM	FOXF1 promotes NPCs and downregulates inflammatory factors in human nucleus pulposus cells via extracellular vesicle delivery	Tang et al. (2021)
	102 ± 8 nm	miR-15a	MMP-3	EVs-miR-15a promotes chondrogenic differentiation of NP-MSCs by downregulating MMP-3 via PI3K/Akt and Wnt3a/β-catenin axes	Zhang et al. (2021d)
CESCs	-	miR-125-5p	NPCs,ECM	CESC-EVs-miR-125-5p promotes autophagy, inhibits apoptosis and ECM degradation in NPCs	Chen and Jiang, (2022)
	-	Sphk2	NPCs	Lenti-Sphk2-Exos can regulate autophagy in NPCs by activating PI3K/AKT signaling pathway *in vitro* and *in vivo*	Luo et al. (2022)
USCs	49.7 ± 7.3 nm	MATN3	ECM	MATN3 in USC-EVs mediates beneficial effects on IVDD.	Guo et al. (2021)
	50–100 nm	-	NPCs	USCs-EVs inhibit stress-induced ER stress and suppress NPCs apoptosis	Xiang et al. (2020)

## 2 Structure of IVD

The IVD is a fibrocartilaginous pad between two adjacent cones that maintains the spine for slight movements (Newell et al., 2017). The IVD is mainly composed of three parts, namely NP, AF, and fibrocartilaginous endplate (CEP), that are distributed concentrically around the NP. The NP is a gel-like structure composed of water, type II collagen, chondrocyte-like cells, and proteoglycans, accounting for 40–50% of the volume of an adult IVD (Iatridis et al., 1996). Water accounts for 70–90% of the total weight of the NP, and the water content of the NP decreases with age (Antoniou et al., 1996). Fluid pressure within the IVD changes with decreasing water content, leading to a derangement of molecular anabolism in the IVD (decreased proteoglycans, glycosaminoglycans [GAG], aggrecans, and type II collagen and increased type I collagen) (Cao et al., 2022). The increase in matrix metalloproteinases (MMPs) in the ECM disrupts the normal equilibrium structure of the IVD and accelerates IVDD. MMP-3 and MMP-13 are considered key factors in ECM degradation in IVDD (Yao et al., 2016; Liang et al., 2022). There is no neurovascular structure around NPCs, and it is difficult to heal it once damaged. After AF rupture, NP prominence exposes the immune system to an otherwise immune IVD (Ye et al., 2022). Recruitment of inflammatory factors [IL-1β and tumor necrosis factor (TNF)] and immune cells (T cells, macrophages, and natural killer cells) to the IVD accelerates IVDD progression by destroying ECM components while clearing necrotic tissues (Risbud and Shapiro, 2014; Sun et al., 2020; Francisco et al., 2022). Maintaining the integrity of NPCs may avoid the massive destruction of the ECM. Therefore, treatment of damaged NPCs is a potential therapeutic target to delay IVDD progression. Replacing or repairing aging NPCs is the main mechanism by which EVs reverse IVDD (Collin et al., 2011).

The AF is a highly fibrotic tissue surrounding the NP and is mainly composed of a concentric fibrous structure comprising type II collagen near the NP and type I collagen outside (Peredo et al., 2020). In addition to this collagen difference, the tension of the outer AF is 2–3 times greater than that of the inner AF (Eyre and Muir, 1976). The AF protects the integrity of the NP and resists spinal stress. Owing to its avascular nature, low cellularity, and dense ECM composition, the AF rarely heals itself after tissue injury. Therefore, when the AF is damaged, it cannot withstand the pressure of the NP, and the NP protrudes into the spinal canal and compresses adjacent nerve roots, causing radicular pain (Peredo et al., 2020). Excessive mechanical load is the main cause of IVDD, with increased expression of the inflammatory factors IL-1β and TNF-α, which may further accelerate IVDD (Gonçalves et al., 2021; Chang et al., 2022). Induction of AF repair is considered an effective strategy for the treatment of IVDD.

The CEP is a thin layer of hyaline cartilage that separates the IVD from the upper and lower vertebral body bones. Unlike that of the AF and NP, the vascular structure within the CEP is the main source of IVD nutrition (Holm et al., 1981; Feng et al., 2022). Nonetheless, there are currently no reports of IVDD reversal by modulating the CEP in IVDD treatment (Lakstins et al., 2021). IVD regression is associated with inflammation, immune cell infiltration, and neovascularization (Krut et al., 2021). This organizing process exacerbates radicular pain in the microenvironment of the degenerative disc (Freemont et al., 1997). In addition, degenerated NPCs aggravate the degeneration of the CEP through EV formation (Feng et al., 2022). CEP denaturation may impede nutrient transport, resulting in failure to maintain material and metabolic balance in the NP (Wang et al., 2018). Therefore, regulation of the NP, AF, and CEP may provide potential targets for IVDD treatment.

Owing to the unique structure of avascular and tightly surrounded by AF around NPs, oral or systemic administration does not enable drug transport into NPCs. Although stem cells exist around IVD, the microenvironment around IVD cannot effectively induce stem cells to differentiate into NPCs (Li et al., 2021a). EVs inherit the advantages of their parental MSCs, which can modulate inflammatory and immune responses, and exert proliferative and antioxidant effects (Priyadarshani et al., 2016). Intra-IVD injection of MSC-EVs can transfer miRNAs and other substances carried by them to NPCs, replacing or repairing damaged NPCs and regulating the ECM components around NPCs to promote their growth (Collin et al., 2011).

## 3 Extracellular vesicles

### 3.1 Generation of extracellular vesicles

EVs are a heterogeneous population of membrane vesicles secreted by all living cells and are grouped into three broad categories based on their subcellular origin and size: apoptotic bodies, membrane vesicles (MVs), and EVs (Veerman et al., 2019). EVs transfer cell membrane and cytoplasmic proteins, lipids and RNAs, and play an important role in maintaining cellular function and tissue homeostasis in multicellular organisms (Raposo and Stoorvogel, 2013; van Niel et al., 2018). EVs represent a new form of intercellular communication, promoting cell proliferation, differentiation, and angiogenesis and inhibiting apoptosis and inflammation to mediate tissue repair (Chang et al., 2021). Cells release different types of endosome- and plasma-membrane-derived membrane vesicles, termed EVs and MVs, respectively, into the extracellular environment (Raposo and Stoorvogel, 2013). MVs are 100–1,000 nm in diameter and are released from cells by direct budding from the plasma membrane (Lee and Kim, 2021). EVs and apoptotic bodies originate from nanoscale vesicles formed by inward budding of the endosomal membrane and are secreted into the extracellular milieu when MVBs fuse with the plasma membrane (Urabe et al., 2020). EVs and MVBs are produced by healthy cells as part of regular membrane turnover and exocytosis. In contrast, apoptotic bodies arise from outer membrane blebbing in cells undergoing apoptosis (El Andaloussi et al., 2013). Apoptotic bodies are 100–5,000 nm in diameter, whereas EVs are much smaller vesicles, approximately 30–150 nm in diameter (Li et al., 2021b). Once EVs are released by cells, they may be taken up by recipient cells to deliver molecules, such as proteins, lipids, and RNA, that they carry to target cells (Veerman et al., 2019). Internalization of EVs by recipient cells relies on a fusion mechanism between the vesicle and plasma membranes and is mediated mainly by clathrin-mediated endocytosis, caveolin-dependent endocytosis, micropinocytosis, and phagocytosis (Kwok et al., 2021). Membrane proteins and membrane receptors also play important roles in EV transmission. Owing to the size and number of MVs and exosomes, relatively pure single structures are usually not available for these vesicles. Therefore, the International Society for Extracellular Vesicles has uniformly named these membrane structures EVs (Théry et al., 2018).

### 3.2 Advantages of extracellular vesicles

#### 3.2.1 As a carrier

EVs are vesicles produced by cells. Compared to polymer biomaterials, EVs have unique advantages as carriers. Their negatively charged surface is intrinsically stable in the circulatory, while enabling EVs to escape attack by monocytes owing to the surface expression of CD47 (Elsharkasy et al., 2020). Additionally, EVs are membrane structures that change their shape to pass through natural tissue and cellular barriers, such as the blood–brain barrier (Chen et al., 2016). The contents of an EV may be affected by factors, such as the type of cell source, physiological state of donor cells, and external stimuli (Kwok et al., 2021). Packaging of goods by EVs is mainly via exogenous and endogenous loading (Gupta et al., 2021). Endogenous loading mainly relies on genetic engineering technology to transform cells before EVs are produced and express or incorporate the mRNA or protein of the cargo into cells. When cells produce EVs, the cargo is wrapped and encapsulated into EVs as the EVs are secreted (Aubertin et al., 2021). This mode of delivery protects the cargo from degradation during transportation and ensures EV integrity. The cargo encapsulation efficiency and loading capacity of EVs depends on the active components of the cargo and its physicochemical properties (Piffoux et al., 2021). However, endogenous loading is only suitable for biomolecule encapsulation, not for the reproducible incorporation of chemically synthesized drugs (Rankin-Turner et al., 2021). Exogenous loading typically involves incorporation of cargo into EVs by various methods, including co-incubation, sonication, thermal shock, and electroporation (Armstrong et al., 2017). Co-incubation is a simple method of encapsulation, and mother cells may be passively loaded by co-incubation with molecular organisms. Packaging efficiency is related to the surface charge and characteristics of the carrier (Rankin-Turner et al., 2021). Sonication results in higher encapsulation efficiency than co-incubation. Sonication relies on ultrasonic waves to temporarily destroy the membrane structure, enabling the encapsulation of cargo into EVs regardless of surface charge and cargo properties (Sato et al., 2016). Electroporation enhances membrane permeability and enables passage of exogenous molecules by applying a transient electric field; in this method, loading efficiency is inversely proportional to the size of the loaded molecules (Heiser, 2000). EVs are secreted by cells *in vivo* and have natural advantages over polymer materials as carriers.

#### 3.2.2 Targeting after transformation

Although EVs are produced by cells and widely present in body fluids, intravenous EVs are cleared in the liver and spleen (Lener et al., 2015). Transformation of EVs results in EV chemotaxis to different locations *in vivo*. This is attributed to the presence of lipids and proteins on the EV surface that are acquired from the cell of origin during their biogenesis (Ou et al., 2021). EV distribution *in vivo* is mainly determined by different cell sources, routes of administration, and targeting (Wiklander et al., 2015). Cells of different origins have cell homing at different locations in the body; for example, immune cells preferentially target immunologically active sites, and this homing is species-independent (Wiklander et al., 2015). Macrophage-secreted EVs target areas of inflammation *in vivo* (Kalluri and LeBleu, 2020). Exosomes expressing the Tspan8-α4 complex are most readily taken up by endothelial and pancreatic cells (Rana et al., 2012). EVs are internalized by receptor cells dependent on ligand-receptor binding (Kwok et al., 2021). Therefore, engineering EVs to express specific ligands on their surfaces is beneficial to improve their targeting properties (Yong et al., 2020). Chemical modification and genetic engineering are widely used methods for EV membrane modification (Zhang et al., 2021b). Inserting a gene encoding a targeting protein into a donor cell, and secretion of protein in exosomes by the donor cell may achieve the corresponding targeting ability (Luan et al., 2017). Elevated expression of chemokine (C-C motif) ligand 18 on tumor cell-derived EVs promoted EV internalization by tumor cells expressing chemokine (C‒C motif) receptor 8 (Berenguer et al., 2018). Chemical modifications also endow EVs with unique targeting capabilities, whereas the biocompatibility of EVs reduces the risk of systemic toxicity commonly found in other nanomaterials.

#### 3.2.3 Avoiding side effects of cell therapy

The ability of stem cells for self-proliferation and direct differentiation has played an important role in tissue engineering and regenerative medicine (Hade et al., 2021; Scala et al., 2021). However, clinical application of stem cells have certain challenges. For example, inadequate retention and survival of MSCs at the site of administration hinders their therapeutic efficacy, as demonstrated in an osteoarthritis study (Arshi et al., 2020). Exogenous stem cells are not conducive to survival and proliferation in the ischemia and inflammatory microenvironment and may even lead to the death of many cells in the body (Zhao et al., 2021). Inflammatory factors released by dead cells also cause peripheral inflammatory responses that are often detrimental to the survival of peripheral cells (Yuan et al., 2021a). Changes in the microenvironment induce dramatic changes in stem cell behavior (Li et al., 2021c). EVs are produced by cells in a paracrine manner, and their production is a normal physiological process. Compared to stem cells, EVs avoid the side effects of cell therapy and play additional roles as carriers, remodeling, and signaling between cells. Stem cell-secreted EVs may replace stem cell-based therapies in various injury and disease models (Hade et al., 2021). MSC-EVs themselves exhibit anti-inflammatory effects, promote the polarization of macrophages to the M2 type, and provide basis for tissue repair (Arabpour et al., 2021). Therefore, EVs reduce adverse reactions by retaining the regenerative ability of stem cells. Additionally, EVs have a low immunogenicity compared to stem cells and will not cause immune rejection in the body (Elahi et al., 2020). Therefore, the development of EVs for tissue engineering and regenerative medicine applications is of great significance.

### 4 Applications of various extracellular vesicles in slowing down intervertebral disc degeneration progression

MSC transplantation has shown great promise in regenerative medicine (Liu et al., 2021). MSCs are multipotent adult stromal cells obtained from the bone marrow, umbilical cord, fat, and placenta (Herman et al., 2021). The application of bone marrow-derived mesenchymal stem cells (BMSCs) in IVDD has been reported. BMSC transplantation into the IVD resulted in the proliferation and differentiation into NP-like cells, a significant increase in viability and autophagy levels, a decrease in rat NPC apoptosis, and the subsequent induction of synthesis of new ECM (Loibl et al., 2019; Shi et al., 2021). Intravenous infusion of MSCs only transports a small number of MSCs to the damaged site, and most MSCs are cleared by the body (Mezey, 2011). EVs produced by MSCs via a paracrine manner are the most important mediators of the therapeutic effects of MSCs (Witwer et al., 2019).

### 4.1 BMSCs

**FIGURE 1:**
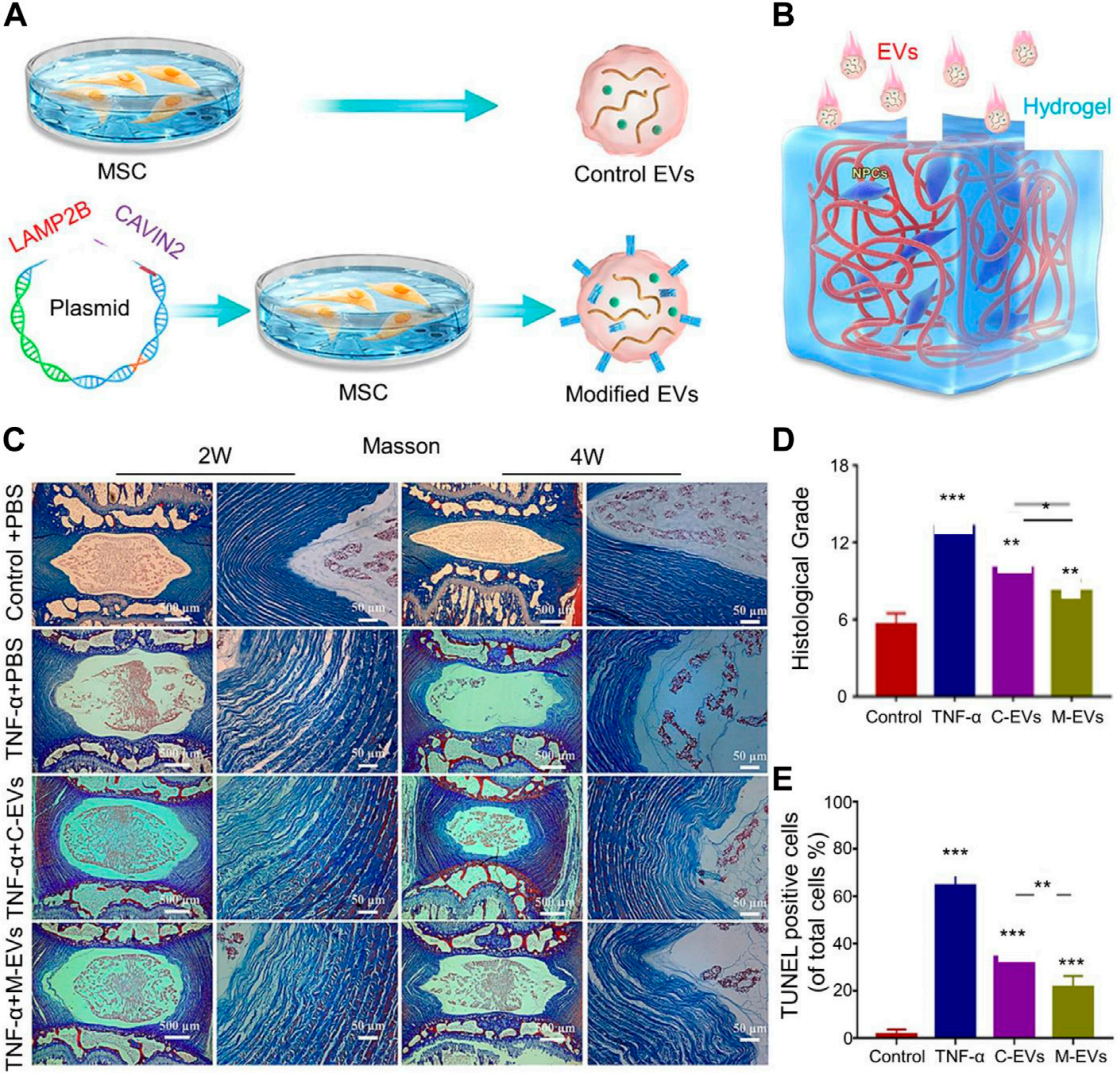
**(A)** Schematic diagram of the extracellular vesicle (EV) engineering process modified by Cavin-2. **(B)** Schematic illustration of EV treatment of intervertebral disc degeneration (IVDD) in an *in vitro* alginate hydrogel model. **(C)** Masson staining histological analysis at 2 and 4 weeks of treatment shows the morphology of IVDs in different treatment groups. **(D)** Histological grade based on the scale of disc degeneration in different groups. **(E)** Terminal deoxynucleotidyl transferase (TdT) dUTP Nick-End Labeling (TUNEL) staining shows the apoptosis of different groups. Reproduced with permission from (Liao et al., 2021).

Excessive oxidative stress and inflammation are the main causes of early IVDD. Mitochondrial production of reactive oxygen species (ROS) increases the number of NOD-, LRR- and pyrin domain-containing protein 3 (NLRP3) inflammasomes around the IVDD (Zhang et al., 2017). The NLRP3 inflammasome is an essential component of the innate immune system, mediating caspase-1 activation and IL-1β secretion that results in cellular damage (Kelley et al., 2019). Apoptosis of NPCs is an important factor causing IVDD; apoptosis of NPCs causes water loss, decreased osmotic pressure, and changes in the quantity and distribution of collagen (Yang et al., 2009). Xia et al. demonstrated that BMSC-EVs inhibited ROS production in NPCs and NLRP3 inflammasome activation and attenuated NPC apoptosis in a rabbit IVDD model (Xia et al., 2019). BMSC cells were cultured *in vitro*, and the supernatant was collected after centrifugation to obtain MSC-EVs. The diameter of MSC-EVs was approximately 50–130 nm, and they were internalized by NPCs. MSC-EVs provided mitochondria-related proteins to NPCs to promote the recovery of mitochondrial function. Additionally, MSC-EVs slowed IVDD progression by reducing the expression of matrix metallopeptidase 13 (MMP-13) in the ECM. To further demonstrate that MSC-EVs reduce the expression of NLRP3 bodies and caspase-1 in NPCs, NPCs were treated with the proinflammatory factor TNF-α to increase the expression of NLRP3 bodies and caspase-1 in NPCs *in vitro* (Liao et al., 2021). Liao et al. have engineered modified MSC-EVs using gene-editing technology to increase their internalization by NPCs (Liao et al., 2021). The vector-expressing Cavin-2 was transfected into the *Lamp2b* gene in MSCs (the Lamp2b protein in the EV membrane) to express Cavin-2 in the EV membrane (Figure 1A). Cavin-2 is involved in caveolae-mediated endocytosis, which increases EV internalization by damaged NPCs. The effects of Cavin-2-modified EVs on NPCs were validated in an alginate hydrogel-simulated IVD *in vitro* (Figure 1B). Injection of EVs into the IVD at 2 and 4 weeks demonstrated that EVs promoted the repair of broken AF in the IVD (Figures 1C,D). EVs also significantly reduced the amount of apoptosis (Figure 1E). In addition to inflammatory factors, reduction of mitochondrial membrane potential by compression caused NP apoptosis (Ding et al., 2012). Hu et al. demonstrated that BMSCs-EVs alleviated compression-mediated NP apoptosis by inhibiting oxidative stress (Hu et al., 2021). *In vitro* experiments demonstrated that BMSCs-EVs had a protective effect on mitochondrial membrane potential and avoided compression-induced mitochondrial damage in NPCs. ROS levels of NPCs in the BMSCs-EV group were significantly lower than those in the compression group alone. Additionally, the accumulation of advanced glycation end products (AGEs) in the NP induced inflammation, metabolic dysfunction, and endoplasmic reticulum (ER) stress and promoted apoptosis of NPCs (Song et al., 2018). MSC-EVs alleviated AGE-induced ER stress by activating the protein kinase B (AKT) and extracellular signal-regulated kinase signaling pathways *in vitro*, thereby reducing NPC apoptosis in IVD progression (Liao et al., 2019; Xiao et al., 2022).

Autophagy is different from apoptosis. Apoptosis is a type of programmed cell death, whereas autophagy is a protective mechanism whereby cellular components are sequestered into lysosomes, which can digest these substrates and recycle them to generate new cellular structures (D’Arcy, 2019). In an *in vitro*-simulated IVD experiment, Zhou et al. co-incubated BMSCs and NPCs in a medium depleted of oxygen and glucose to mimic the oxygen-deficient state of IVD (Shi et al., 2021). BMSC-EVs also provided nutrients to NPCs and increased the expression of miR-155, thereby promoting the expression of autophagy marker proteins LC3 and Beclin-1 and formation of autophagosomes (Chen et al., 2019; Shi et al., 2021). MiR-155 inhibited apoptosis of NPCs by inhibiting the expression of caspase-3 (Wang et al., 2011; Zheng et al., 2013). Decreased autophagy capacity of NPCs was detected after knockdown of miR-155. miRNA expression was reduced in apoptotic NPCs, whereas EVs contained abundant miRNAs. Therefore, using EVs to provide miRNA to NPCs to inhibit apoptosis is a main mechanism by which EVs reverse IVDD (Chen et al., 2010). Cheng et al. demonstrated that BMSCs-exosomes inhibited apoptosis of NPCs by activating signaling pathways (Cheng et al., 2018). MiR-21 in MSC-EVs inhibits NPCS apoptosis by targeting phosphatase and tensin homolog (PTEN) through the phosphatidylinositol 3-kinase (PI3K)-AKT pathway, a phenomenon not observed in fibroblast-EVs; this indicates that EVs derived from different mother cells have different functions. Bone marrow MSC-EVs deliver miRNAs through different pathways to inhibit NPC apoptosis (Table 1) (Zhu et al., 2020a; Zhu et al., 2020b; Cui and Zhang, 2021; Wen et al., 2021; Yu et al., 2022a; Yu et al., 2022b; Sun et al., 2022). Another study showed that the pH in degenerated IVD decreased from the normal 7.0–7.2 range to 6.5–5.7. Compared to that in normal IVD pH conditions, the number of cells in BM-MSCs was reduced by 40 and 80% after 3 days of culture at pH 6.8 and 6.5, respectively (Lyu, 2022). Therefore, the acidic environment in aged NPCs is not conducive to the growth of MSC-EVs.

In IVDD, BMSCs are the most studied intervention among MSCs from other sources. Cytokine profiling of BMSCs, however, revealed intensive production of proinflammatory cytokines IL-6 and IL-8 (Kiselevskii et al., 2021), which may contradict the report that MSCs induce anti-inflammatory effects (Marimuthu and Pushpa Rani, 2021). In addition, BMSCs aid the expression of vascular endothelial growth factor (VEGF) to promote angiogenesis under hypoxic conditions (Archacka et al., 2021). These factors are not conducive to IVDD recovery. However, there is currently no report on VEGF following IVDD treatment with BMSC-EVs.

### 4.2 Placenta-derived/umbilical cord-derived MSCs (PLMSCs)

PLMSCs have more unlimited differentiation potential and a lower immunogenicity than BMSCs (Miao et al., 2006). Overexpression of chondrogenic transcription factors in PLMSCs accelerated their differentiation potential into chondrogenic progenitors, resulting in better homing, integration, and differentiation into NPCs of IVD (Khalid et al., 2022). miR-4450 targets NPCs to induce apoptosis, and its overexpression increases apoptosis and the inflammatory responses of NPCs and reduces ECM components to promote IVDD progression (Yuan et al., 2020). Zhang et al. collected placentas immediately after cesarean section and transfected the anti-miR-4450 gene (antagomiR-4450) into PLMSCs for culture (Yuan et al., 2020). After purification by ultracentrifugation, PLMSCs-EVs with inhibited miR-4450 gene expression were obtained, with diameters ranging from 30 to 150 nm. NPCs were co-cultured with PLMSCs-EVs, which transferred antagomiR-4450 into NPCs, to alleviate the NPC damage. The decreased expression of caspase-3 and MMP-13 and increased gait frequency were detected on the 42nd day in mice. Gait frequency is a powerful tool to evaluate exercise capacity in laboratory animals and clinical patients (Jarchi et al., 2018). The increase in gait frequency illustrates the recovery of motor capacity in mice. In conclusion, PLMSCs-EVs attenuated NPCS injury and delayed IVDD injury *in vivo*.

N6-methyladenosine is the most common endogenous RNA modification (Huang et al., 2018). Methyltransferase (METT) removes miRNA methylation and reduces miRNA expression. METTL14 exists in NPCs and stabilizes the expression of NLRP3 mRNA. NLRP3 induces inflammation in IVDD and accelerates the apoptosis of NPCs and progression of IVDD (Yuan et al., 2021b). Yuan et al. used a lentiviral vector that targets human METTL14 to carry miR-26a-5p for transfection into human umbilical cord mesenchymal stem cells (HUCMSCs) (Yuan et al., 2021b). HUCMSC-EVs were 65 ± 15 nm in diameter. HUCMSC-EVs provided miR-26a-5p to prevent NPC apoptosis by targeting METTL14 and inhibiting the production of METTL14 and NLRP3. Additionally, HUCMSC-EVs promoted the expression of collagen II and aggrecan in the ECM to delay IVDD progression (Qi et al., 2019).

PLMSCs-EVs release angiopoietin to stimulate angiogenesis (Komaki et al., 2017). While IVDs are avascular structures, and angiogenesis is not conducive to maintaining IVD structures, whether PLMSCs-EVs induce angiogenesis in IVD has not been reported. Furthermore, compared to BMSCs, PLMSCs and UC-MSCs are not easily available and have ethical issues that limit their application.

### 4.3 Adipose-derived mesenchymal stem cells (ADSCs)

**FIGURE 2:**
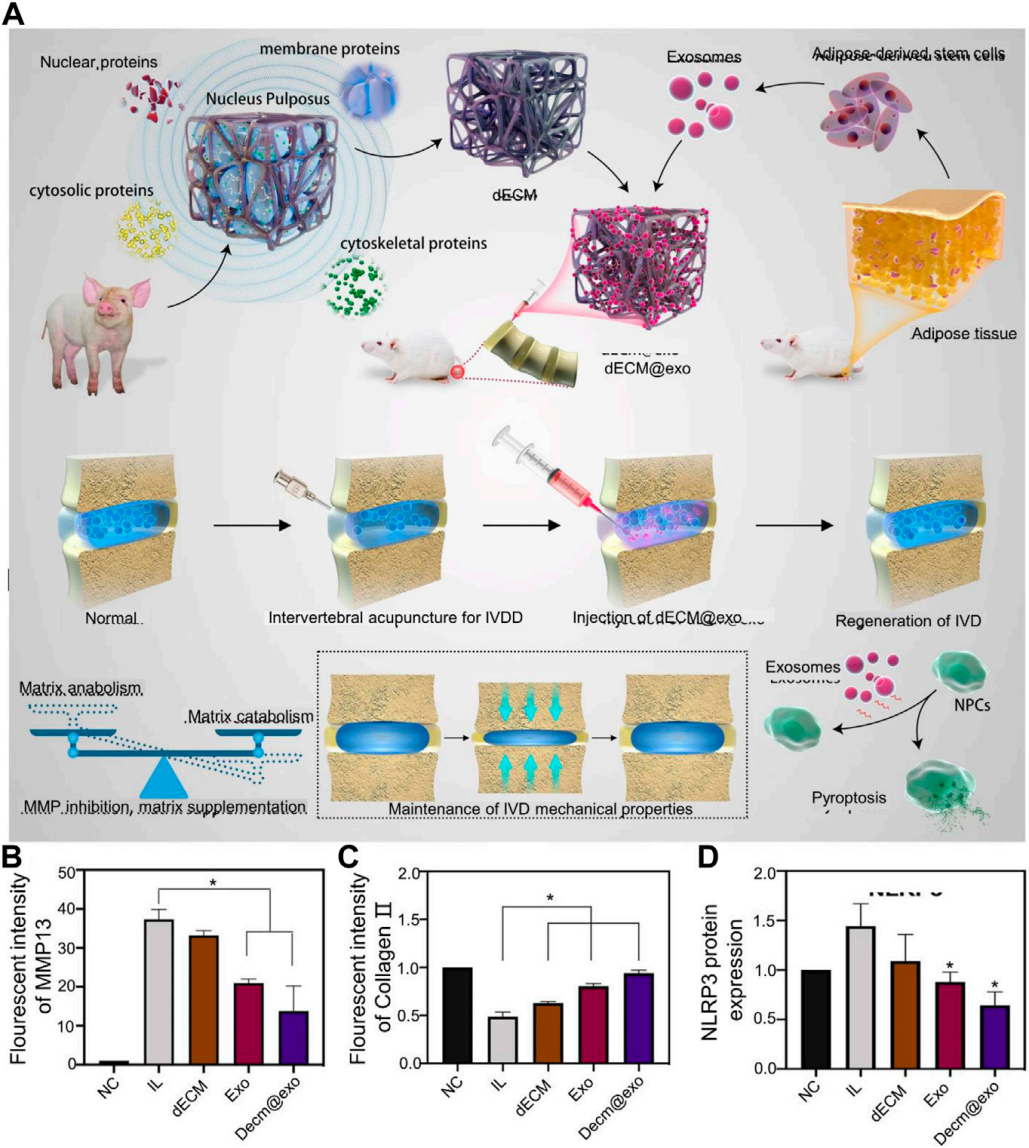
**(A)** Schematic diagram of the preparation mechanism of dECM@exo. **(B)** Quantitative analysis of the fluorescence intensity of matrix metallopeptidase 13 (MMP-13). **(C)** Quantitative analysis of the fluorescence intensity of collagen 11 in each group. **(D)** Quantification of NOD-, LRR- and pyrin domain-containing protein 3 (NLRP3) expression. Reproduced with permission from (Xing et al., 2021).

The main advantages of ADSCs over other sources of MSCs include abundance of tissue sources, ease of tissue collection and cell isolation, and their therapeutic potential (Bunnell, 2021). ADSCs are accessible, and there is an abundance of tissue that may be obtained in large quantities without affecting body function. Compared with other MSCs, ADSC-derived extracellular nanovesicles significantly promoted the mRNA expression of chondrocyte genes (collagen-II, aggrecan and Sox-9) in human NP cells (Zhang et al., 2021c). ADSC-EVs enter NPCs and the AF to reduce the expression of inflammatory factors and MMPs *in vitro* (González-Cubero et al., 2022). Zhang et al. demonstrated that ADSCs-EVs increased the migration and proliferation of NPCs and suppressed their inflammatory activity (Zhang et al., 2021c). ADSC-EVs detected the expression of the collagen-II gene in NPCs on the first day of co-incubation with NPCs. Decreased levels of inflammatory factors IL-1α, IL-1β, and TNF-α were detected on the seventh day. However, the low number of EVs generated from ADSCs with a short duration of action cannot sustainably support IVDD treatment. Xing et al. developed a decellularized extracellular matrix (dECM) hydrogel using porcine NP matrix for the delivery of ADSC-EVs (Figure 2A) (Xing et al., 2021). Porcine NP matrix was regenerative, and dECM@exo had a porous structure that retains glycosaminoglycans (GAGs) to remove 99% of DNA components. ADSC-EVs, with a diameter of 30–150 nm and a zeta potential of −32.7 mV, were stably bound to negatively charged groups in the ECM. *In situ* injection of dECM@exo supplemented ECM leakage in NPCs and provided an environment for the growth of NPCs. *In vitro* experiments confirmed that dECM@exo was able to gradually release most EVs for up to 28 days at 37°C. dECM@exo downregulated MMP-13 (Figure 2B) and upregulated collagen II (Figure 2C) to promote ECM synthesis and maintain the stability of IVDD. Additionally, dECM@exo reduced the expression of the NLRP3 inflammasome and suppressed the inflammatory response of IVD (Figure 2D).

### 4.4 Induced pluripotent stem cells (iPSCs)

Although MSC-EVs have made progress in relieving NPC aging and promoting ECM synthesis, harvesting MSCs from bone marrow and adipose tissues is invasive. In addition, the decreased proliferative potential and therapeutic efficacy of MSCs during *in vitro* expansion limits the application of MSC-EVs (Larson et al., 2010). iPSCs are a subset of stem cells that can be reprogrammed from any tissue type in the body (Cartwright et al., 2005). They have a unique ability to proliferate indefinitely and show totipotency *in vitro*. Compared with MSCs, EVs can be obtained in large quantities from iMSCs. iPSCs have a high proliferation rate and may be cultured for 40 passages *in vitro* and reprogrammed from any tissue type *in vivo* (Ohnuki and Takahashi, 2015). Senescence of NPCs plays a critical role in the pathological progression of IVDD (Jiang et al., 2013); aged NPCs have a decreased ability to increase proliferation and decompose ECM components, thus aggravating IVDD progression. The expression level of Sirt6 was reduced in senescent NPCs, and Sirt6 repaired broken DNA duplexes to protect cells from senescence (Tian et al., 2019). Sun et al. used EVs isolated from iPSCs for the treatment of IVDD (Sun et al., 2021). The diameter of iMSCs-sEVs was 80–200 nm iMSCs-sEVs were injected into a rat IVDD model, and miR-105-5p from iMSCs-sEVs activated the Sirt6 pathway in aging NPCs at the eighth week, thereby delaying the growth of NPCs, aging, and IVDD progression. iPSCs differentiated into NP-like cells *in vitro* in the presence of a specific medium and addition of growth factors (Chen et al., 2013; Tang et al., 2018). This study addressed the pitfalls of low availability of autologous NPCs in adults. However, this study only conducted *in vitro* experiments, and further confirmation of whether the NP-like cells synthesized *in vitro* may inhibit NPC degeneration and induce NPC regeneration *in vivo* is needed.

### 4.5 Nucleus pulposus stem cells

**FIGURE 3:**
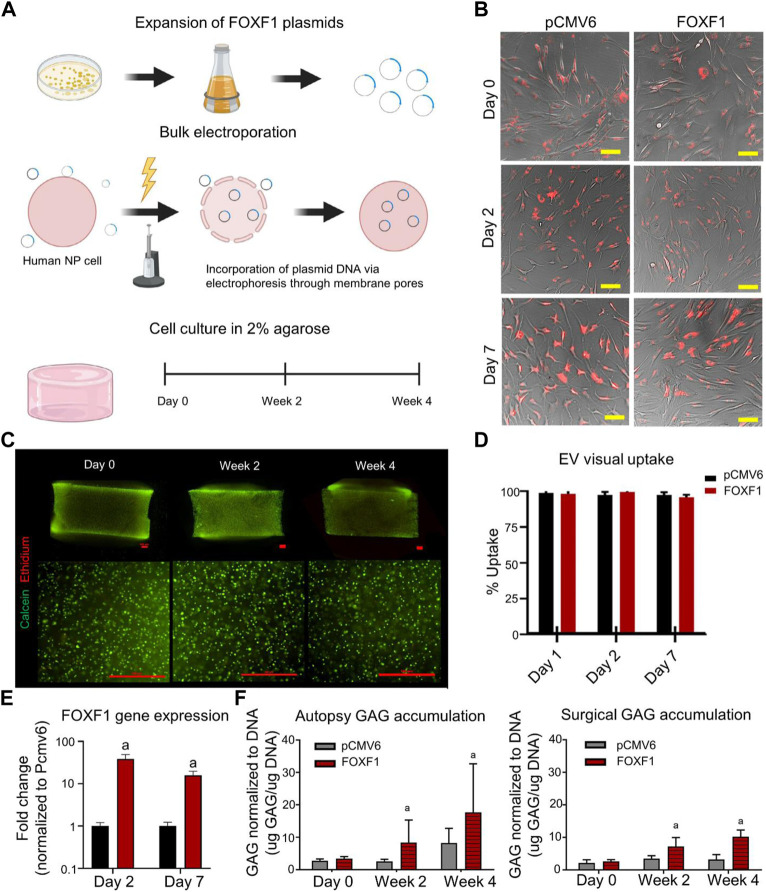
**(A)** Schematic representation of *in vitro* reprogramming of human nucleus pulposus (NP) cells by bulk electroporation of Forkhead-FOXF1). **(B)** Brightfield images of NP cells co-cultured in monolayers with PKH26-stained pCMV6 and FOXF1 extracellular vesicles (EVs, red), respectively, at 0, 2, and 7 d. **(C)** Representative images (bottom) of 4 × (top) and 10 × images (bottom) of cell-embedded three-dimensional (3D) agarose gels at days 0, 2, and 4 in culture (scale bar: 500 μm). Live cells are stained green (calcein-AM) and dead cells are stained red (ethidium-homodimer). **(D)** Quantified visual uptake by the percentage of cells with EVs over the total number of cells. **(E)** FOXF1 expression of FOXF1-EV-treated human NP cells in monolayers normalized to pCMV6-EV-treated cells. **(F)** Normalized to DNA content of autopsy and surgical human NP cells in 3D culture at 0, 2, and 4 weeks. Reproduced with permission from (Tang et al., 2021).

NP-MSCs have been found in degenerative and normal IVD tissues. NP-MSCs were significantly less inhibited under acidic *in vitro* conditions and expressed significantly higher levels of proteoglycans and collagen II than other MSCs (Zhang et al., 2021d). Forkhead-box F1 (FOXF1) is rarely expressed in degenerated NPCs. Therefore, transferring the FOXF1 transcriptional gene into degenerated NPCs to convert degenerated NPCs to healthy NPCs may be a therapeutic modality for IVD (Zhang et al., 2021d). FOXF1 is a healthy NP-specific marker involved in the regulation of cell differentiation, growth, and proliferation (Tuteja and Kaestner, 2007). Tang et al. transfected *FOXF1* into a plasmid and transfected the plasmid into NP-EVs through electroporation (Figure 3A) (Tang et al., 2021). A total of 95% EVs containing *FOXF1* were endocytosed by NPCs after 7 days (Figures 3B,D) and rapidly expressed in NPCs (Figure 3E). During *in vitro* culture for 4 weeks, transfection of FOXF1 did not affect cell survival (Figure 3C). After *FOXF1*-containing EVs were endocytosed by NPCs, upregulated expression of GAG in the IVD was detected (Figure 3F). NPMSCs may be isolated from degenerated IVD and non-degenerative IVD; however, non-degenerative IVD-derived NPMSCs have a stronger clonogenicity, higher proliferation rate, and higher migration rate than metamorphically derived NPMSCs (Jia et al., 2017). NPMSCs transplanted into IVDD that differentiate into NPCs to replace damaged NPCs are the best treatment method for IVDD. However, hypoxia regulation in degenerated IVD is not conducive to maintaining the number of NPMSCs (Tian et al., 2020). Compared to other types of MSCs, NPMSCs can upregulate the expression of hypoxia-inducible factor (HIF), collagen II, and proteoglycan genes (Marimuthu and Pushpa Rani, 2021). HIF is a transcription factor of cellular response to hypoxia, and NPCs express HIF-1α to maintain cellular energy metabolism and the ECM (Tran et al., 2013). HIF also reduces oxidative stress in the endoplasmic reticulum in degenerated discs (Novais et al., 2021). miR-15a targets and downregulates MMP-3 through the PI3K/AKT and Wnt3/β-catenin signaling pathways and increases collagen II and aggrecan levels (Zhang et al., 2021d). Zhang et al. isolated NPMSCs from NP tissues to regulate MMP-3 components in the ECM. EVs were isolated from NPs, and miR-15a was transfected into NP-EVs to provide miR-15a to NPMSCs (Zhang et al., 2021d). EVs containing miR-15a have a diameter of 102 ± 8 nm and increased the proliferation ability of NPMSCs after being taken up by NPMSCs. This study confirmed that anti-miR-15a inhibited collagen II and promoted the expression of MMP-3. Therefore, miR-15a derived from NP-EVs interacted with NP-MSCs to maintain the stability of IVDD.

### 4.6 Cartilage endplate stem cells (CESCs)

**FIGURE 4:**
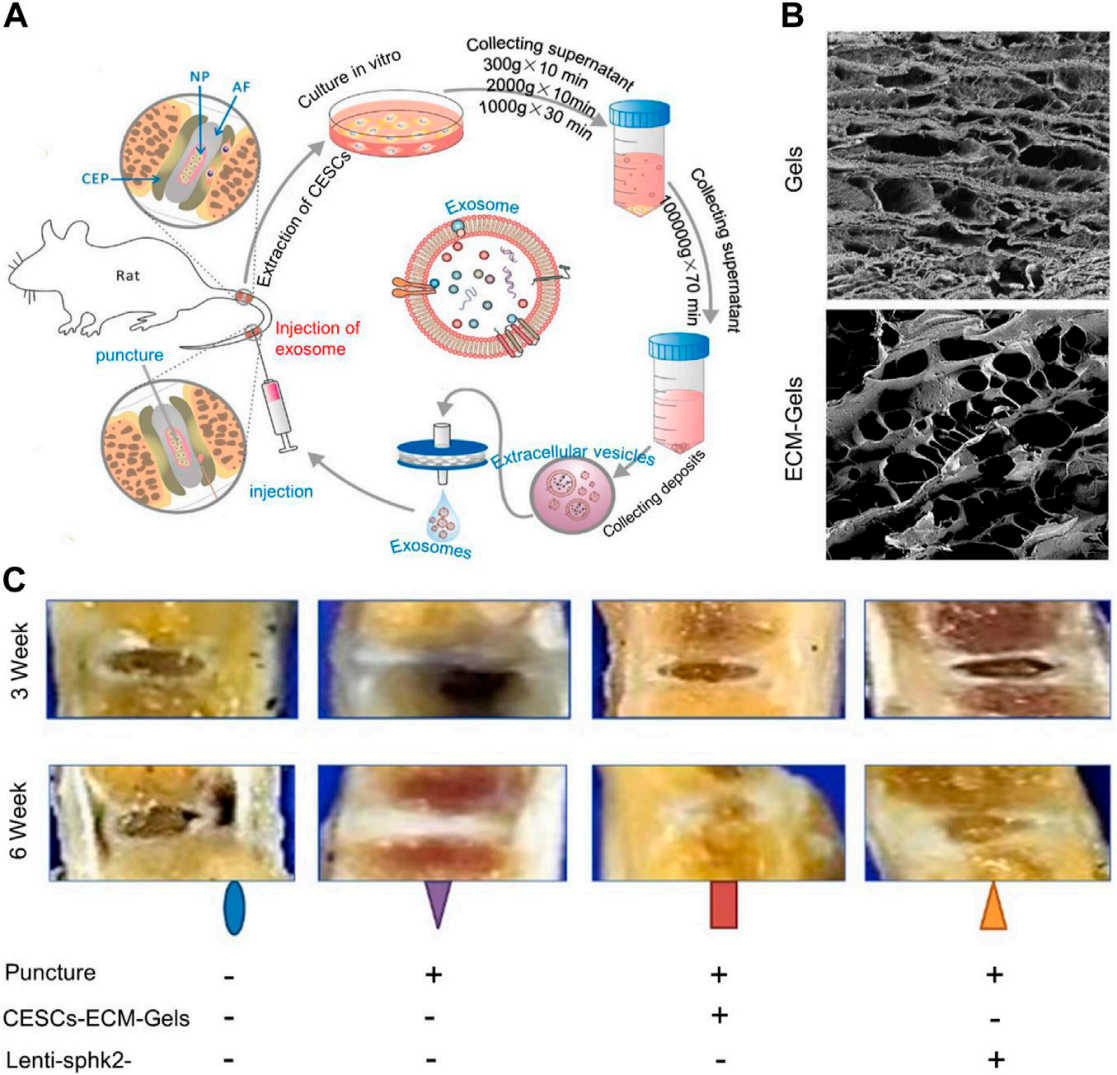
**(A)** Flowchart of exosome extraction and treatment of intervertebral disc degeneration (IVDD). **(B)** Scanning electron microscopy images of hydrogels with or without extracellular matrix (ECM) modification of costal cartilage. **(C)** Morphology of IVD tissue at 3 and 6 weeks after treatment with ECM gel and Lenti-Sphk2-extracellular vesicles (EVs) in the control group. Reproduced with permission from (Luo et al., 2022).

Approximately 5% of MSCs cells in the IVD are produced by AF, NP, and CEP cells (Harris et al., 2018). The distribution of spine-derived MSCs varies widely, with 18,500–61,875 MSCs isolated from 0.8 mm of CEP. CESCs are cell populations isolated from the CEP that differentiate into osteoblasts, adipocytes, and chondrocytes (Luo et al., 2021a). Compared to MSCs from other sources, CESCs are better at osteogenesis and chondrogenesis (Liu et al., 2011). They activate the HIF-1α/Wnt pathway via autocrine EVs to promote CESC migration and transdifferentiation into NPCs (Luo et al., 2021b). Normal tissue-derived CESCs are better at attenuating NP apoptosis by activating autophagy via the PI3K/AKT pathway than degenerating tissue-derived CESCs (Luo et al., 2021a). This effect was attenuated after blocking the PI3K/AKT pathway. Dong et al. secreted EVs from CESCs to carry miR-125-5p for targeting NPs (Chen and Jiang, 2022). Entry of miR-125-5p into NPCs downregulated the expression of caspase three and MMP-13 and upregulated that of GAG and collagen II. To increase the stability of EVs *in vivo*, Luo et al. developed a costal cartilage ECM hydrogel to deliver CESCs and transfected the *Sphk2* gene into EVs using a lentiviral vector (Figure 4A) (Luo et al., 2022). Overexpression of Sphk2, a native protein, enhanced autophagic flux and suppressed NPC senescence. The hydrogels doped with the ECM of the costal cartilage had large voids, which were favorable for the growth of CESCs (Figure 4B). CESCs in the hydrogels released CESC-EVs and penetrated the AF within 72 h. The released EVs delivered Sphk2 to NPCs, enhanced autophagic flux, and inhibited NPC senescence. These EVs also reduced expression of IL-6 and MMP-13 in the IVD. In a mouse tail IVD model, Lenti-Sphk2-CESC-loaded ECM-Gels inhibited IVDD (Figure 4C). Although the hydrogel delivery of CESCs provides a theoretical basis for the treatment of IVDD, many questions remain to be resolved. For example, the properties of hydrogels and the time they may be maintained *in vivo* have not been confirmed, nor has the ability of EVs released from CESCs to be maintained *in vivo*. Additionally, Sphk2 is mainly expressed in the cytoplasm, which may inhibit senescence and promote proliferation, whereas its expression in the nucleus has the opposite effect (Wang et al., 2019). Stromal cell-derived factor-1 that is released in NPCs and degenerated CEPs also aggravates CESCs and ECM damage, thus promoting IVDD (Zhang et al., 2018).

### 4.7 Human urine-derived stem cells (USCs)

USCs are stem cells obtained from human urine with multi-differentiation potential. Compared to MSCs from other sources, USCs are safe, non-invasive, widely sourced, and most importantly, do not involve ethical issues (Ji et al., 2017). Although their origin is unknown, USCs are likely derived from glomerular parietal cells (Wu et al., 2021). The lack of costimulatory molecules and MHC-II markers on USCs suggests a lower immunogenicity. During cell proliferation, telomeres gradually shorten and decrease, which reduces their proliferative capacity. Telomerase maintains the length of telomeres and the ability of the cell to proliferate. USCs have abundant telomerase activity and maintain their proliferation rate (Huang et al., 2022). Matrilin 3 (MATN3), a member of the matrilin protein family, is mainly distributed in chondrocytes and plays an important role in the synthesis of cartilage ECM. A reduction in its expression level may lead to IVDD. Zhang et al. demonstrated that USC-EVs enriched in MATN3 protein promoted NPC proliferation and ECM synthesis, thereby alleviating IVDD (Guo et al., 2021). NPCs could be endocytosed by NPCs within 3 h of co-incubation with USCs-EVs. USCs-EVs provide MATN3 to NPCs and promote ECM synthesis. After MATN3 knockout, USCs-EVs promoted the proliferation of NPCs and decreased ECM synthesis. Additionally, USCs-EVs changed the conformation of the transforming growth factor *β* (TGF-β) precursor complex by secreting MATN3, thereby promoting TGF-β activation. Activation of the TGF-β/SMAD pathway promoted collagen type II expression in the ECM. In another study, USCs-EVs inhibited IVDD by ameliorating endoplasmic reticulum stress (Xiang et al., 2020).

Owing to their uncertain origin, the safety of USCs is an important issue limiting their application. USCs express 571 genes responsible for neuromuscular diseases (Falzarano et al., 2021). The biological characteristics of USCs are not fully understood. USCs may be differentially expressed in various media owing to their different surface markers (Bento et al., 2020). In addition, the growth and proliferation ability of USCs from various aged sources differed. Therefore, side effects from USCs should be examined before their clinical application.

## 5 Challenges of extracellular vesicles in intervertebral disc degeneration

Although EVs play a role in delaying IVDD through intercellular signaling, EVs from various sources have different phenotypes, and EV secretion may be artificially induced by adjusting the composition of the medium. However, some issues remain to be resolved. At present, the mass and stable production of EVs are the main limitations of their clinical application (Luan et al., 2017). The most direct way to scale up production is to increase the number of EVs produced by a single cell, such as through three-dimensional (3D) culture methods or physical or chemical stimulation (Grangier et al., 2021). Compared to 2D culture technology, such as the use of hyperflasks or roller bottles as supports, 3D culture technology minimizes operation time, culture period, and cost by increasing the available surface area (Grangier et al., 2021). Obtained EVs are then subjected to ultracentrifugation for purification. The production of EVs may be considered a response of cells to their microenvironment (Staubach et al., 2021). Thus, it is unknown whether changes in the composition of the medium after large-scale culture and physicochemical stimulation may cause EVs to be secreted in a direction that deviates from what is needed. Different EV subpopulations emerge even within EV populations. Even current state-of-the-art techniques cannot completely separate EVs from subpopulations (Qin et al., 2021). Ultracentrifugation is the gold standard for the isolation of EVs; however, it is time consuming and cannot separate larger protein complexes (Momen-Heravi, 2017). Precipitation separates EVs by adding anionic complexes for coprecipitation with positive charges on the EV surface (Rider et al., 2016). However, contamination by co-precipitation of larger proteins and EVs cannot be resolved. Commercial size-exclusion chromatography (SEC) columns have been widely used recently. SEC-separated EVs have intact membrane structure and less contaminants (Tesovnik et al., 2021). However, there is currently no standard for the functional characterization of EVs obtained using this method. In other words, it is unknown whether EVs obtained from mass production undermine their therapeutic effect *in vivo*.

Another major role of EVs is as a carrier to transport substances to target cells. EVs carry DNA, various RNAs, proteins, and lipids of the parent cell after paracrine secretion. EVs are endocytosed by target cells and release their contents into target cells to regulate their function. The membrane structure of EVs makes them a safe delivery carrier. However, the low loading efficiency of EVs limits their application (Luan et al., 2017). At present, the loading methods for EVs include co-incubation, sonication, decompression, and electroporation. However, the loading efficiency of co-incubation is low, and the membrane structure of EVs is disrupted by ultrasonic electroporation. Likewise, there is currently no method for mass loading. Furthermore, how long post-production EVs may be stored *in vitro* and their *in vivo* stability after storage remain unknown. Therefore, it will take some time for EVs to be used clinically.

## 6 Conclusions and Outlook

As the most common degenerative disease of the spine, IVDD seriously affects the physical and mental health of patients. For patients with IVDD, physical therapy only relieves pain but cannot eliminate the root cause of IVDD. After IVDD causes severe pain and neurological symptoms, chiropractors usually remove the IVD or decompress the spinal canal. However, after surgery, IVD remains at a high risk of herniation while aggravating damage to adjacent segments. As a result, surgery causes the patient to endure pain, often despite its high cost. Biomaterial-related tissue engineering and regenerative medicine experiments in animals have reported delay in IVDD progression. The 3D printed bracket provides a certain local pressure. Hydrogels modulate the immune-inflammatory microenvironment around the IVD to slow IVDD progression. However, it will take time before their clinical application.

EVs are an emerging therapeutic modality for IVDD treatment that may address immune rejection and the ethical shortcomings of cell therapy while maintaining its advantages. EVs have been fully demonstrated in recent years as a method of signal transmission between cells and substance carriers to target cells. Furthermore, the role of EVs in regenerative medicine is becoming increasingly prominent. EVs deliver anti-apoptotic mRNAs to NPCs to maintain NP activity, target the ECM to degrade MMPs, and promote collagen II and GAG synthesis to delay IVDD progression. EVs also deliver mitochondria-associated proteins to NPCs to inhibit NLRP3 activation. Methods of blocking apoptosis of NPCs have been demonstrated in preclinical studies. The subpopulation of MSCs-EVs is large, the components are complex, and the differences across batches are difficult to determine; thus, it is difficult to prepare a standard dosage for a particular disease treatment, and individual reactions are unpredictable. Although there is no clinical report on the use of EVs in IVDD, their applications for the treatment of acute coronary syndromes, Parkinson’s disease, and cancer have been explored. We believe that future EVs will contribute to a major breakthrough in slowing the progression of IVDD.
